# Our Daughters Ruined

**Published:** 1856-12

**Authors:** 


### OUR DAUGHTERS RUINED.

WHERE?

At fashionable boarding-schools.

How?

In manner and form to wit:

A young lady in good health was sent to a distant city, to finish her education at a boarding-school of considerable note. In one month she returned, suffering from general debility, dizziness, neuralgic pains, and headache.

It must be a very telling process, which, in a single month, transforms a rollicking, romping, ruddy-faced girl of sixteen, to a pale, weakly, failing invalid. It is not often done so quickly; but in the course of a boarding-school education, it is done thousands of times. Public thanks are due to a correspondent of the *Buffalo Medical Journal*, for the pains he took to ferret out the facts of the daily routine of the establishment, the proprietors of which so richly merit the reprobation of the whole community, both for their recklessness of human health, and their ignorance of physiological law. Said an accomplished lady to us not long since, "My only daughter is made a wreck of—she lost her mind at that wretched school!"

At this model establishment, where the daughters of the rich and of the *aspiring* are prepared for the grave every year, twelve hours are devoted to study, out of the twenty-four, when five should be the utmost limit.

Two hours are allowed for exercise.

Three hours for eating.

Seven hours for sleep.

Plenty of time allowed to eat themselves to death, at the expense of stinting them to the smallest amount of time for renovating the brain, the very fountain of life, upon whose healthful and vigorous action depends the ability of advantageous mental culture, and physical energy.

But what is the kind of exercise which prevails in city boarding-schools? The girls are marched through the streets in double file, dressed violently, of course, so as to inure to the benefit of the proprietors, in the way of a walking advertisement, knowing well enough that a file of young ladies, from the families of the upper ten, would monopolize attention on any thoroughfare, even Wall-street. But what does an hour's prim walk effect, when, conscious of being the cynosure of every eye, they are put on their most unexceptionable behavior, when a good side-shaking, whole-souled laugh would subject the offender to a purgatorial lecture, to be repeated daily, perhaps, for a month? Verily, Moloch has his worshippers in this enlightened age, when parents are found to sacrifice the lives of their daughters, for the reputation of having them at *the* fashionable boarding-school.

